# Surgery of Teat and Udder in Small Ruminants: Lesions, Techniques and Outcomes of 135 Cases

**DOI:** 10.3390/vetsci13020112

**Published:** 2026-01-23

**Authors:** Sebastian A. Mignacca, Benedetta Amato, Maria Costa, Marcello Musicò, Giovanna L. Costa

**Affiliations:** 1 Pathology Division, Department of Agriculture, Food and the Marine, Backweston Campus, W23 VW2C Celbridge, Ireland; 2 Department of Pathology, Bristol Veterinary School, Bristol BS40 5DU, UK; 3 ASP Enna, 94100 Enna, Italy; 4 Department of Veterinary Sciences, University of Messina, 98168 Messina, Italy

**Keywords:** anaesthesia, goats, mammary gland, sheep, surgery, teat, trauma, udder

## Abstract

A retrospective study on 135 cases of teat and udder surgical conditions in 129 small ruminants is described. On 19 repairs of teat lacerations, a primary- and a secondary-intention healing in 13 (68%) and in 4 (21%) cases, respectively, was observed; 2 (11%) had poor response and consequent mastitis. Good outcome and first-intention healing in 100% of the fistula repairs (2 cases), thelectomies (5 cases), teat neoplasm removals (14), and mastectomies (2 cases) were observed. Among 26 teat curettage cases, all 18 (69%) unilateral lesions treatment had a good outcome versus the 8 (31%) with bilateral lesion that suffered definitive relapse. In 67 skin udder neoplasm removals, a primary- and a secondary-intention healing in 59 (88%) and in 8 (12%) cases, respectively, was observed; however, 2 of the latter suffered mastitis. These procedures are associated with a good prognosis, and the percentage of favorable outcomes was high. Wound infections and dehiscence were the main complications observed. More interest in teat and udder surgery on small ruminants should be encouraged, and farmers should be made aware that the animal can often return into production at a reasonable cost; however, their post-operative care is the key to success.

## Simple Summary

Many veterinary pathological conditions affecting the teat and udder can result in chronic poor health and a negative impact on both animal welfare and production, and should require effective surgery to relieve the associated discomfort and pain. While the greater part of the available knowledge refers to bovine conditions, the literature in small ruminants is scant. The clinical presentation, surgical approach and outcomes of 135 pathological teat and udder conditions in 129 sheep and goats are here described. The cases consisted of repairs of teat lacerations and fistulas, thelectomies, teat curettages, removals of skin neoplasms and mastectomies. Overall, interventions were associated with a good prognosis, and the percentage of favorable outcomes was high; on the other hand, wound infections and dehiscence were the main complications observed in cases with poor outcomes. However, the post-operative care was considered the key to success. Repairs of recent teat lacerations represented urgent interventions. Thelectomies or skin neoplasm removals often were simple and plannable. Bilateral stenotic lesions of the canal should not be overtreated. Mastectomies required more technical knowledge but were plannable, thus predisposing the surgery to a good outcome too. Procedures can be easily undertaken on-farm with basic surgical sets and are not particularly challenging, but often require precise anatomic, surgical and anesthesiologic knowledge.

## 1. Introduction

Pathologies of the mammary gland usually lead to loss of milk production, which decreases the economic value of the animals involved [1], and may lead to their premature culling [2].

In small ruminants, teat wounds are more common than in cattle [3], above all in those animals whose very large udders predispose them to trauma [4].

Teat-acquired fistula could be the consequence of deep traumas or could be a negative outcome of previous surgery; sometimes, it could have a congenital origin [5]. Nevertheless, teat fistulas do not occur often in ewes [6].

Obstructions of the milk ejection in “hard milkers” may be due to teat spiders, milk stones, bacterial thelitis, traumas, frost injury, malfunctioning of the milking machines or genetic inheritance [3].

Hyperthelia, or supernumerary teats, is a congenital condition of ancillary teats in addition to the primary teats [7], and its percentage can reach 64.92% in ewes and 30.72% in does [1].

Skin neoplasms of the teat and the udder are quite common, especially in breeds with depigmented skin that are reared in areas largely exposed to UV rays [8]. The majority of these lesions are represented by papilloma, fibropapillomas and squamous cell carcinomas [9].

On the other hand, many udder diseases are characterized by severe and irreversible impairment of organ functions, such as suppurative or gangrenous mastitis [10,11], chronic sclerosing mastitis [12], pendulous udder, parenchymal neoplasms, inappropriate lactation syndrome [13,14] and gynecomastia in bucks [3,15,16]. Thus, affected animals could represent good candidates for surgery.

### Anatomy

In small ruminants, the teat’s wall consists of the skin, a large intermediate layer (mainly represented of connective tissue, smooth muscle and numerous large blood vessels), a very thin submucosa and a mucosa which is composed of two floors of cuboidal epithelium [17]. The orifice (*ostium papillae*) located at the distal end of the teat is internally communicating with the teat duct (*ductus papillaris*), then follows the teat cistern (*sinus papillaris*); this latter is empirically separated from the gland cistern of the relative udder by an annular ring, which contains a large vein that encircles the base of the teat [6,18].

**Figure 1:**
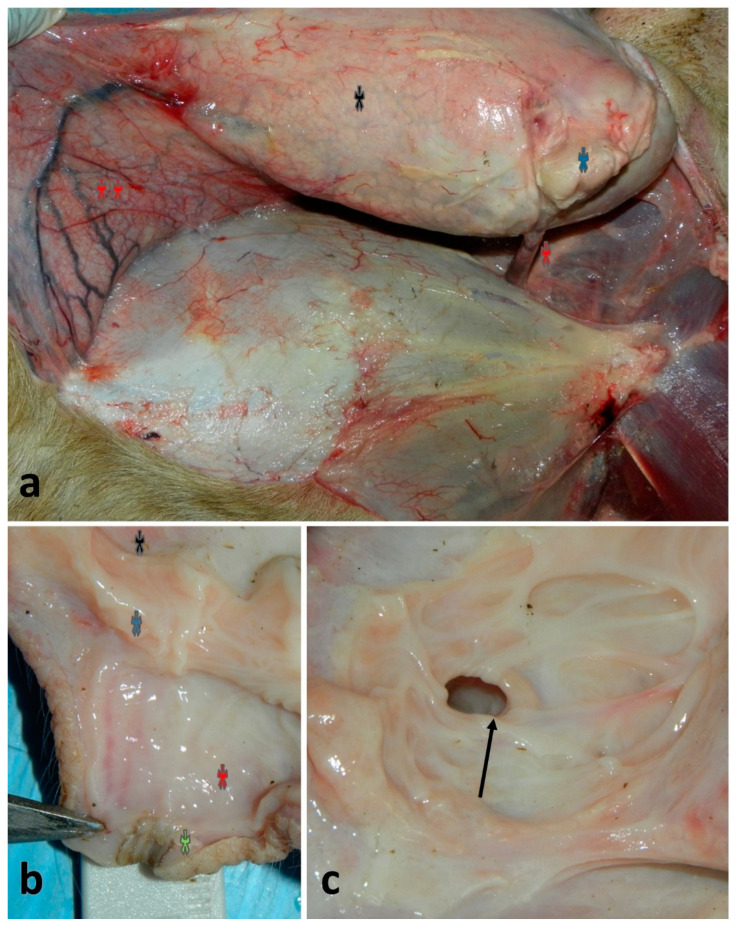
Anatomy of udder and teat in an ovine cadaver: (**a**) specimen showing the anatomical connections between the mammary gland and the abdominal wall, including the mammary lymph-node (*), the overturned mammary gland (*), the mammary artery and vein (*) and the subcutaneous abdominal veins (**); (**b**) longitudinal opening of the teat showing the teat duct (*), the teat cistern (*), the annular ring (*) and the gland cistern (*); (**c**) dorsal view from the gland cistern showing the annular ring (arrow) and the underneath teat cistern.

Each half-udder in small ruminants is formed by the skin, the subcutis, the superficial fascia, an area under this fascia (rich in blood vessels, nerves and lymph vessels), the internal fascia and the mammary parenchyma [18]. Each half of the udder is supplied mainly by the external pudendal artery, that emerges from the inguinal ring and gives a number of branches to the udder and continues cranially as the superficial cranial epigastric artery. At the same time, each half of the udder is drained by a circular venous plexus, which derives mainly from the external pudendal, the subcutaneous abdominal and the perineal veins (Figure 1a–c). The relatively poor blood supply of the small ruminant udder, together with the scarcity of anastomoses, are considered to be predisposing factors for gangrenous mastitis in sheep and goats [19].

The cranial part of the udder is innervated by the ventral rami of iliohypogastric and the ilioinguinal nerves, while the caudal part is innervated by the pudendal nerve [3].

Although most of the knowledge of mammary gland and teat surgery regards bovine cases [17], surgical treatments are also often applicable in small ruminants and aim to salvage the integrity and the function of the teats and mammary glands, especially in pets and in valuable animals. However, there are several gaps in knowledge for some surgical injuries and their prognosis, management and long-term follow-up in sheep and goats, such as teat and udder skin cancers or the curettage of unilateral and bilateral streak canal stenosis.

This article describes the on-farm surgical procedures and outcomes of teat and udder lesions in dairy small ruminants reared in Sicily (Italy). The surgeries here described were performed for free with a double aim: (i) to increase data on small ruminant surgery; (ii) to collect skin neoplasms for further investigations.

## 2. Materials and Methods

Treatment involved 135 cases in 129 animals. To be precise, 19 repairs of teat wounds, 2 repairs of fistulas, 26 curettages of teat cisterns and of their orifices, 5 thelectomy (teat amputations), 14 removals of neoplasms from the teat skin, 67 removals of neoplasms from the udder skin and 2 mastectomies are discussed (Table 1).

For all these interventions, sedation, anesthesia, surgical field preparation, surgery and post-operative management are described in each section. Usually, the animals were manually and adequately restrained by the owners in lateral recumbency or standing position.

Throughout the surgery, respiratory and heart rates and, when appropriate, non-invasive systolic pressure by a multi-parameter monitor (Leonardo model, AMI Italia srl, Milano, Italy) were monitored; any variation equal or more than 20% of the normal values was considered animal discomfort.

When the difference between a good outcome and a poor outcome of the surgeries was analyzed, the authors considered there to be a good outcome when, beside the good wound healing, teat function and milk yield and/or animal welfare were well maintained/restored (i.e., in case of mastectomy milk production was not preserved but the animal was spared by premature culling). On the other hand, a poor outcome was indicated when the previously mentioned parameters were not preserved/restored.

The following-up of all the cases was obtained via telephone or with clinical examination during the on-farm referral, which was spread from six months to over two years.

For the present study, no animal experiments were performed, and all the surgical procedures refer to animals routinely enrolled in the veterinary practice.

### 2.1. Repair of Teat Wounds

**Figure 2:**
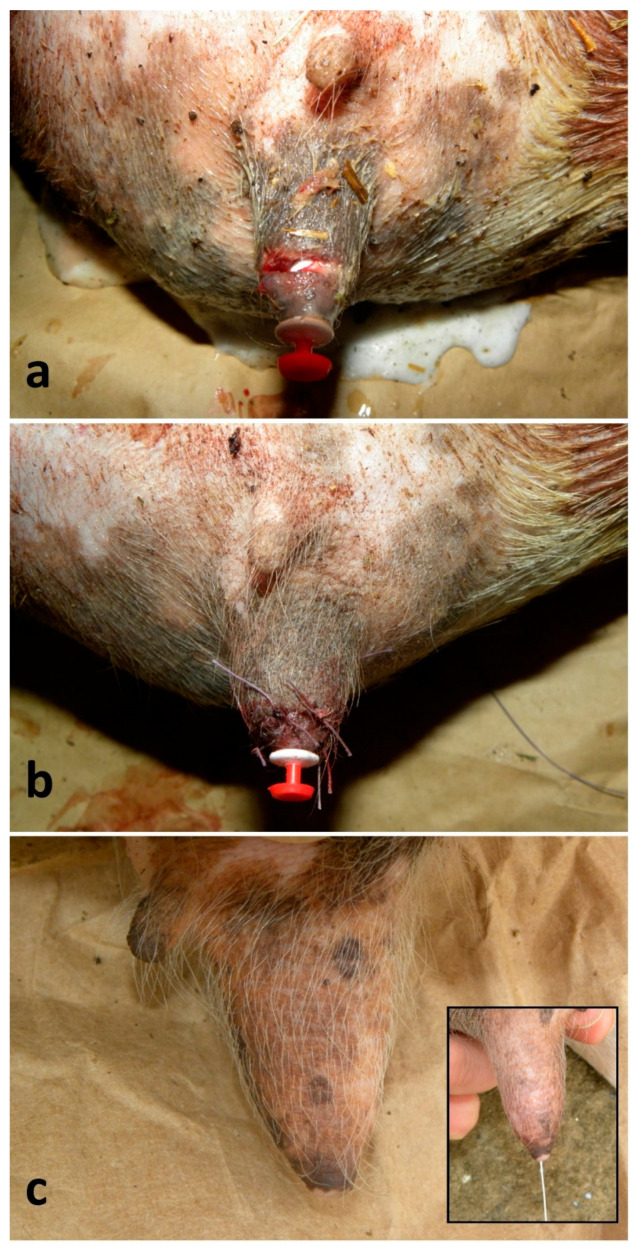
Repair of teat wound in a lactating ewe: (**a**) recent, semi-circular and penetrating teat wound with a teat cannula inserted; (**b**) closure completed by a simple interrupted suture of the muscle and of the skin (the underneath suture of mucosa and submucosa is not shown); (**c**) outcome after 6 months with healed wound and complete resolution of the functions.

Seventeen ewes and two does were treated. Wounds were consequent to traumas (i.e., bites, cuts) and had different shapes and sizes. Only the lesions penetrating through the wall and into the teat sinus were selected for this study. When the orifice was involved, surgery was excluded due to the very poor prognosis. The surgery was performed within 24 h from the injury, but, when possible, the owner also started the antibiotic and NSAID therapy before the intervention. Animals were adequately restrained, the area was disinfected with a chlorhexidine-based disinfectant and a ring block infiltration with 2–6 mL of lidocaine 2% (Lidocaine^®^—Esteve, Milan, Italy) around the teat base was performed. The teat was clamped at its base by Kelly forceps or by a simple or double overhand knot with a strip of gauze bandage, and a sterile plastic cannula (Bovivet teat plugs cannula^®^—Kruuse, Langeskov, Denmark) was inserted inside the streak canal before suturing (Figure 2a). A continuous, non-penetrating suture of mucosa and of submucosa, using a round-bodied needle and a 3-0 or 4-0 polydioxanone suture (PDS) (Polysorb^®^—Covidien, Milan, Italy), was used, followed by a simple interrupted suture of the muscle and of the skin, using a triangular needle and a 1-0 or 2-0 PDS (Polysorb^®^—Covidien, Milan) (Figure 2b). Animals were gently hand-milked once a day through the cannula, and the cannula was replaced every 48 h. Post-operative care included 1–2 ° g of dihydrostreptomycin associated with 1–2 ° million IU of penicillin G procaine (Penistrepto 20/20^®^—Virbac, Milan, Italy) (IM, once a day -OD-, for 6 days) and 10–15 ° mg of flunixin (Finadyne^®^—MSD, Rome, Italy) ^¥^ (IM, repeated after 12 h when necessary). Moreover, the surgical wounds were treated with tetracyclines spray (Neospray Caf^®^—Gellini, Milan, Italy) for 3–5 days.

### 2.2. Repair of Fistulas

Two ewes, one with congenital fistula and one with acquired fistula (due to a non-treated wound) underwent surgery. Both lesions were located at the wall of the teat. Animals were adequately restrained, and the area was disinfected with a chlorhexidine-based disinfectant. Local anesthesia by 2 mL of lidocaine, using an inverted “V” block infiltration, was performed. The teat was clamped with Kelly forceps and, thereafter, a sterile plastic disposable cannula was introduced into the orifice. A metallic surgical thin probe (Ø 2mm × 15cm) was introduced inside the fistula to facilitate dissection, and a small elliptical full-thickness incision was carried out around it. The sutures and the post-operative care were the same as the teat wound repairs.

### 2.3. Curettage of the Teat Cistern and of the Orifice

Twenty-two ewes with contracted sphincter, called “hard milkers”, were treated. Lesions were unilateral in 18 animals, and bilateral in 4. Lesions were represented by partial stenosis of the teat sinus, but in the bilateral lesions the teats orifice seemed more affected than the sinus. Animals were adequately restrained, and the teat orifice was disinfected with a chlorhexidine-based disinfectant. When necessary, the teat was emptied and desensitized with an intraluminal injection of 1–2 mL of lidocaine 2%, simultaneously applying a finger pressure around the teat base for few minutes. After that, a lubricated and disinfected Hug teat punch—Ø 2–4 mm (Erbrich Instrumente GmbH, Tuttlingen, Germany) to remove debris—was introduced. In 5 cases with unilateral lesions, 12 and 24 h later, a new intervention to obtain an optimum milk flow was necessary. After each treatment, 0.1–0.2 g (1/2—full tube) of cloxacillin benzathine suspension (Orbenin lattazione^®^—Zoetis, Rome, Italy) was inoculated via intracanalicular, and a sterile plastic cannula was inserted.

### 2.4. Thelectomy

**Figure 3:**
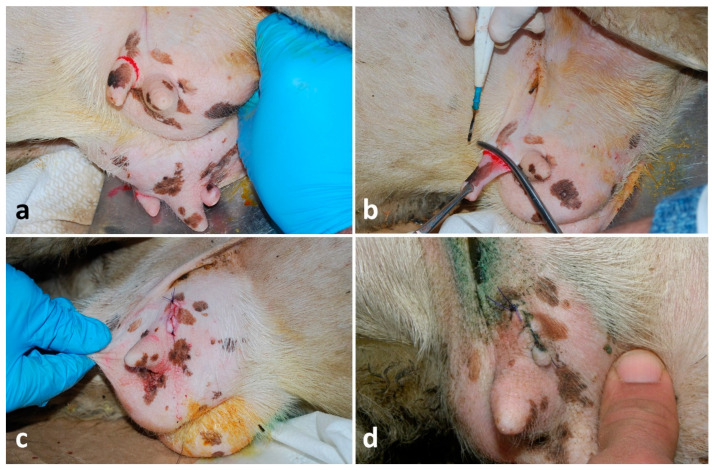
Thelectomy in a lactating ewe: (**a**,**b**) identification and application of a forceps at the base of the desensitized supernumerary teat and its removal using an electrosurgical spatula; (**c**) closure of muscle and skin by a simple interrupted suture (the underneath suture of mucosa and submucosa is not shown); (**d**) good outcome on the 16th day post-surgery.

One lactating ewe and one young goat, both bilaterally affected by supernumerary teats, and one lactating ewe with a neoplasm complicated by myiasis at the teat orifice, were treated. Animals were adequately restrained, and the area was disinfected with a chlorhexidine-based disinfectant. The gland was emptied and treated with 0.2 g of cloxacillin benzathine of antibiotic via intracanalicular. A ring block infiltration with 4–6 mL of lidocaine 2% around the teat base was performed, forceps were applied at the base of the teat and the dissection was performed by using an electrosurgical spatula blade (KLS Martin ME MB 2—KLS Martin Group, Tuttlingen, Germany). Leaving forceps in place, two layers of suturing were applied. One included mucosa and submucosa, and it was a continuous, inverting and non-penetrating suture with 2-0 PDS; instead, muscle and skin were closed using a simple interrupted pattern with 0 PDS (Polysorb^®^—Covidien, Milan, Italy) (Figure 3a–c). The post-operative care was the same as teat wound repairs.

### 2.5. Removal of Neoplasms from the Teat Skin

**Figure 4:**
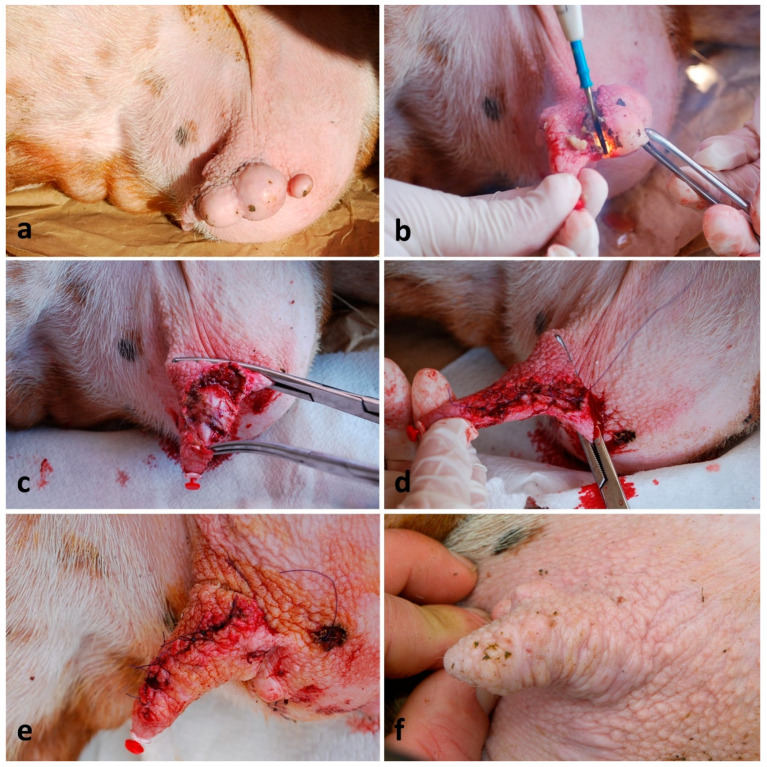
Teat neoplasm removal in a lactating ewe: (**a**–**c**) steps of a multiple papilloma removal located alongside the teat using an electrosurgical spatula; (**d**) closure of subcutis with a simple running suture; (**e**) closure of skin using a simple interrupted suture; (**f**) good outcome after 3 months.

Fourteen ewes were treated. Animals were adequately restrained, the area was disinfected with a chlorhexidine-based disinfectant, and a cannula was introduced into the teat. Anesthesia was performed by injecting 4–8 mL of lidocaine 2% approx. 5–10 mm beside and external to the incision line, and the teat was clamped at its base. In some cases, dissection and tumor removal were performed using a scalpel blade, while, in other cases, an electrosurgical spatula blade was used. The subcutis was sutured with a continuous suture pattern by using a 3-0 PDS, while the skin was sutured with a simple interrupted suture, by using a 2-0 or 1-0 PDS (Figure 4a–e). The post-operative care was the same as the teat wound repairs.

### 2.6. Removal of Skin Neoplasms from the Udder

**Figure 5:**
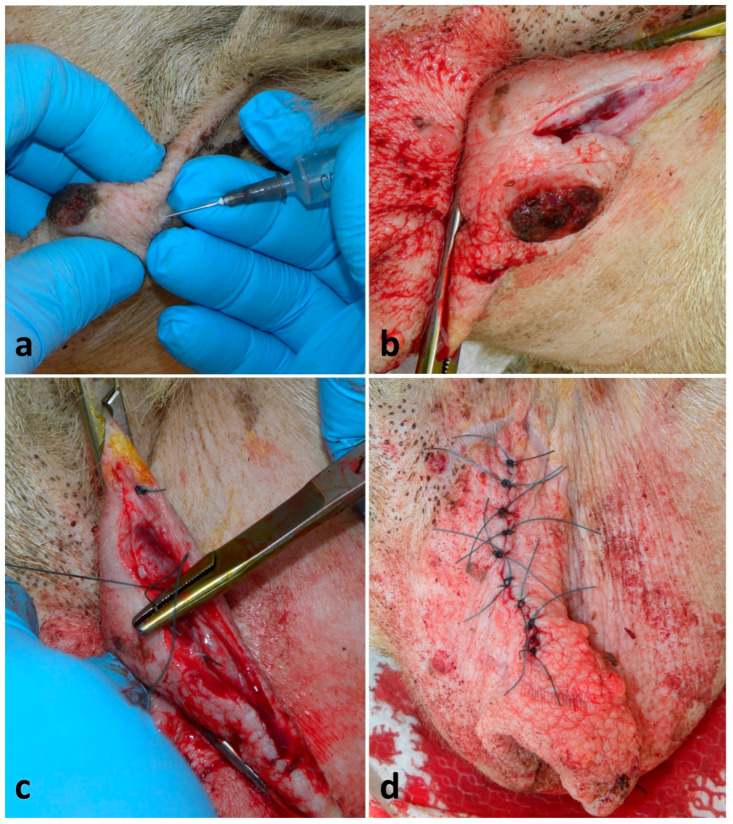
Removal of a tumor located at the lateral surface of the udder in a dry ewe: (**a**) local anesthesia surrounding the lesion; (**b**) clamping using Kelly forceps, dissection and removal of the lesion including a sufficient surrounding tissue using a scalpel blade; (**c**) closure of subcutis with a simple running suture; (**d**) closure of skin with a simple interrupted suture.

Sixty-seven ewes underwent surgery. In most of the animals, lesions were located on the caudal aspect of the udder. Animals were adequately restrained, the area was disinfected with a chlorhexidine-based disinfectant and desensitized by using 10–30 mL of lidocaine 2%, approximatively 1–3 cm far from the incision and into the subcutaneous tissue under the neoplasm. Skin was incised with a scalpel blade all around the tumor, drawing an ellipse encompassing approximately 1–2 cm from the outer portion of the lesion, while the subcutis was dissected by using Mayo scissors; blunt dissection was usually preferred to minimize intraoperative hemorrhage. Vessels were identified, clamped and ligated if necessary. Subcutis was closed with a simple running suture (overlock) by using a 2-0 or 1-0 PDS. Skin was closed either via continuous suture by using a 1-0 or 0 PDS in 45 animals, or via a simple interrupted suture by using a 0 PDS in the other 22 animals (Figure 5a–d). The post-operative care was the same as the teat wound repairs, but, when necessary, the flunixin administration lasted 3 days.

### 2.7. Mastectomies

**Figure 6:**
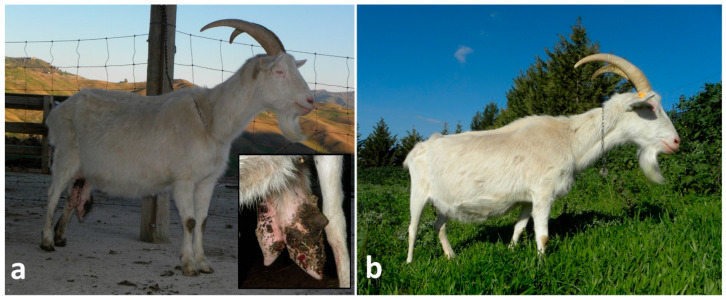
Mastectomy in a doe: (**a**) dry pregnant Saanen doe with multifocal to coalescing severe squamous cell carcinoma at the udder that requested a total mastectomy; (**b**) the doe on the 38th day post-surgery.

Two pluriparous Saanen does affected by severe and diffuse cutaneous neoplasms on the udder (Figure 6a) underwent mastectomy. The two animals were withheld feed and water for 24 h and 12 h, respectively, before the surgery. Sedation was obtained by administering 2 mg/kg IM of tiletamine/zolazepam (Zoletil^®^—Virbac, Milan, Italy) ^¥^, associated with 0.05 mg/kg IM of xylazine 2% (Rompun^®^—Dechra, Turin, Italy) ^¥^. They were placed in right lateral recumbency and firmly restrained in the “horse castration” position (upper hind limb pulled cranially), and an IV catheter was placed. The udder was disinfected with a chlorhexidine-based disinfectant. About 20 mL of anesthetic solution containing 15 mL of lidocaine 2% previously mixed with 5 mL of physiological saline solution was injected in both the ischiorectal fossae (10 mL each side), while, at the base of the udder, in proximity of the supramammary lymph nodes and along the incision line, 50 mL of anesthetic solution, containing 35 mL of lidocaine 2% previously mixed with 15 mL of physiological solution, was deposited. The surgical field was prepared according to the standard procedure. Specifically, the fleece surrounding the udder was clipped, and the skin was thoroughly cleaned using a mild detergent, followed by rinsing with sterile water; the area was then disinfected with a povidone–iodine or chlorhexidine solution and dried. To facilitate handling and minimizing contamination, the udder was placed into a disposable rectal examination glove, and sterile drapes were positioned around the site. Skin was incised at about 4–5 cm below the dorsal edge of the udder, extending the incision line through the lateral suspensory ligament in an elliptic manner. Therefore, two skins flaps (one on each side) were created. Both the external pudendal arteries and veins were identified and ligated. The entire half-udder was isolated from the abdominal wall by an electrosurgical spatula blade while all the vessels of medium and large caliber were isolated and ligated. The same procedure was applied to the contralateral gland, and the organ was removed along with the respective lymph nodes. Subcutis was closed by using a simple running suture with a 2-0 PDS, while skin was sutured via a continuous interlocking suture and simple reinforcement knots by using a 0 PDS. Simple knots were applied to obliterate dead spaces and subcutaneous pockets, especially proximally to the external inguinal ring. A latex Penrose drain was placed at the most ventral aspect of the dead space.

During the surgery, discomfort in both patients was observed, and 1 mg/kg of tramadol (Altadol^®^—Formevet, Milan, Italy) ^¥^ IV was administered. Post-operative care included 2 g of dihydrostreptomycin and 2 million UI of penicillin G procaine (IM, OD, for 10 days), associated with 10 mg of flunixin (IM, OD, repeated for 3 days). Moreover, the surgical wound was treated with tetracyclines spray for 7 days.

° Depending on the animal body weight. ^¥^ Administered off-label, but in accordance with recommended withdrawal periods, and ensuring regulatory compliance.

## 3. Results

For the 19 reconstructions of teat lacerations, a primary-intention healing of 13 (68%) cases and a secondary-intention healing of 4 (21%) cases were observed. Overall, good healing was already appreciated on the 7th day post-intervention; in cases where the trauma involved the distal portion of the teat, but the orifice was intact, the functional integrity of the organ was always preserved (Figure 2c). The four cases resolved by secondary intention required additional 10–14 days to obtain same degree of healing. Two (11%) animals suffered dehiscence, thelitis and mastitis.

The two (100%) sheep treated for fistula showed the same post-operative fate of the teat lacerations with a primary-intention healing.

On 26 teat curettages, all 18 (69%) unilateral lesions were successfully resolved, even though, in 5 cases, a new intervention was necessary, showing then a mild-to-moderate local teat inflammation. On the other hand, all eight (31%) teats of the four ewes with bilateral lesions showed a poor outcome; in these four animals, a primary resolution was obtained only in one case, while, for the other three, an additional intervention at 12, 24 and 36 h post-surgery was necessary. All four animals showed a relapse within six months and, in two of them, a total bilateral obliteration of the treated teats at the next lactation was observed.

Thelectomy showed a complete resolution on the 18th day (±4 days) (Figure 3d) after the surgical removal of all five (100%) teats.

Treatments of the 14 (100%) teat neoplastic lesions achieved great results in their healing process already on the 7th day and a complete healing on the 21st day (Figure 4f), but this depended on the shape and size of the wound.

**Figure 7:**
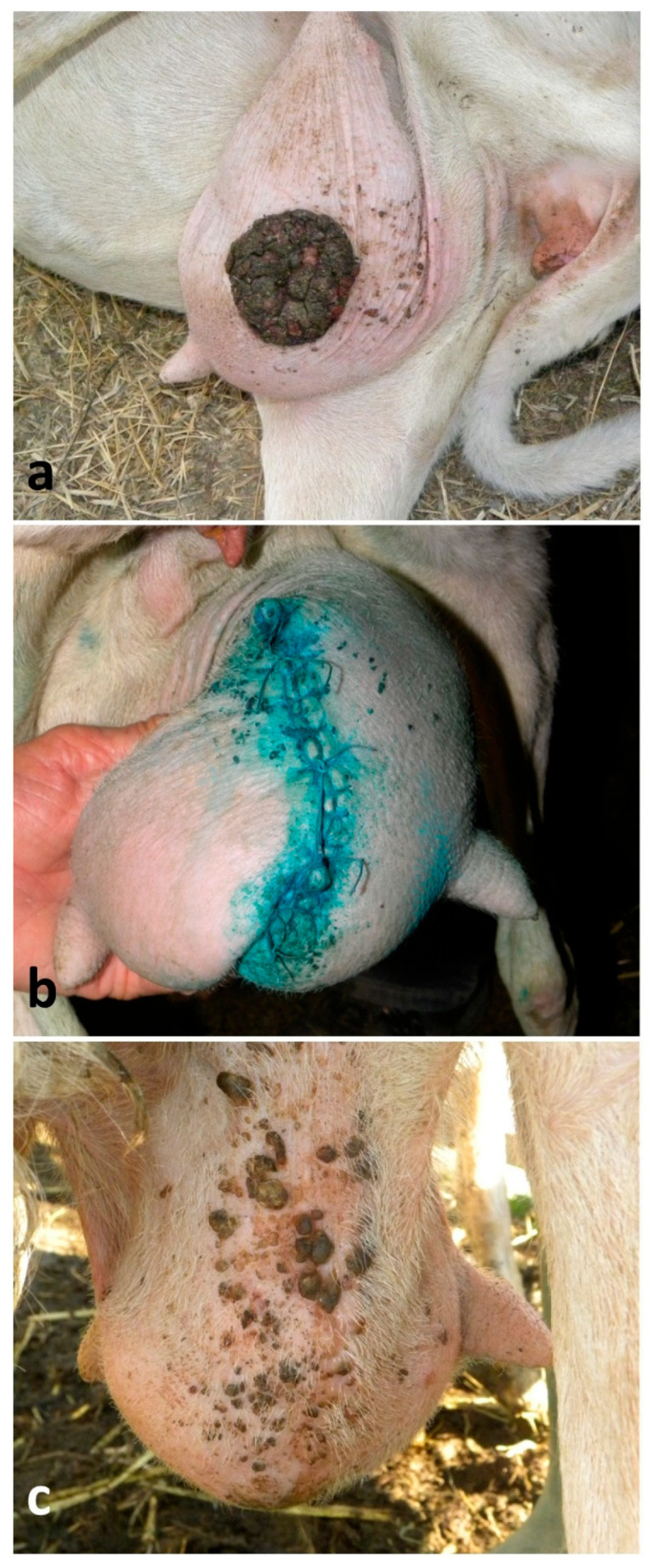
Good healing course of a skin neoplasm removal from the udder in a lactating ewe: (**a**) skin tumor in an ewe (note the numerous small papillomas surrounding the larger mass); (**b**) closure of skin using a simple interrupted suture, and good healing on the 5th day post-surgery—closure of the subcutis is not shown; (**c**) outcome after 6 months that shows healing by primary intention (note the growth of small multifocal papillomas).

**Figure 8:**
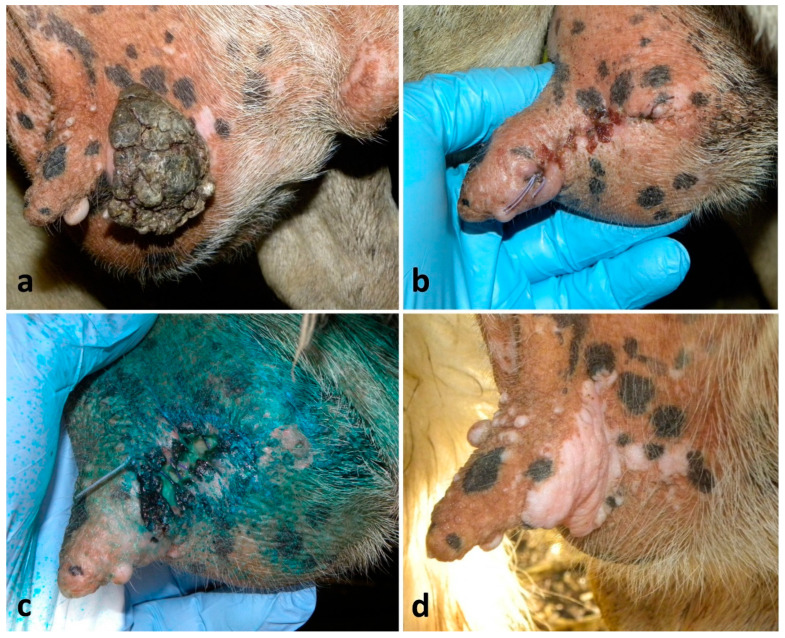
Poor healing course of a skin neoplasm removal from the udder in a lactating ewe: (**a**) single skin tumor located at the cranial aspect of the udder; (**b**,**c**) outcome on the 2nd and on the 7th day, respectively, showing infection, poor healing and dehiscence of the continuous suture due to the incorrect post-operative care by the owner; (**d**) outcome after 6 months showing a healing by second intention and a large scar (the teat functionality was preserved).

Regarding the removal of the 67 neoplastic lesions of the udder skin, 59 (88%) of them showed primary-intention healing, and the healing times ranged between 17 and 26 days (Figure 7a–c). However, the continuous sutures showed greater traction than the simple interrupted ones, and teats showed a permanent slight curvature as result. Healing depended on the extension of the surgical wound, but above all, on the owner’s compliance. Indeed, in eight (12%) cases animals, were not correctly treated with antibiotics during the post-operative care, showing wound inflammation on the 2nd day, and suppuration and dehiscence on the 5th–7th day and healing by secondary intention with extended scar (Figure 8a–d); two of them suffered mastitis.

Regarding the mastectomies, follow-up was evaluated daily. In the days following surgery, moderate subcutaneous edema and discharges through the drainage points were observed. The drainage was removed 1 week post-surgery. The suture and the surrounding skin were slightly swollen and painful and ketoprofen-based ointment—25 mg/g (Fastum gel^®^—Menarini, Firenze, Italy) in derogation—was applicated. Fourteen days after, a suppurative focus in the anchor point from which flowed a mild amount of smelly pus lasting approx. 1 week was observed in a doe. Thirty-eight days after the intervention (±6 days), the follow-up revealed a complete healing of the suture in both animals (Figure 6b).

## 4. Discussion

With the increasing culture of small ruminants being kept as pets, veterinarians are facing new diagnostic and treatment challenges that go beyond the traditional health management of the conventional food animal species kept for a commercial purpose [20]. Moreover, many pathological conditions affecting the teat and the udder can result in chronic poor health and a negative impact on both animal welfare and production, and should require effective surgical treatments to eliminate the associated discomfort and pain [14,20] and restoring/maintaining production when possible, which all represent key priorities.

Teats are prone to various degrees of injuries because of direct trauma, particularly at the end of gestation and the start of lactation, mainly in dairy breeds [14]. This reduces their ability to function efficiently, and predisposes them to teat fistulae, mastitis or sloughing of the udder [2]. Although non-penetrating teat lacerations often do not require suturing, the full-thickness ones should be sutured since their surgical treatment is associated with good prognosis [2]. However, complications such as the delay in the healing process and fistula formation may be recorded. The success in most of the cases in this study was attributed to the fact that wounds were usually treated within 24 h, adequate medical therapy was administered as soon as possible and because only the cases without an orifice involved were intentionally selected for surgeries. Similarly, the good outcome of the fistula repair cases included in this work (although only two cases) was likely also achieved by the plannability of intervention and the fact that wound margins were neat and without loss of tissue. In the rest of the cases, the poor outcome of teat surgery was likely caused by lack of owner compliance.

Regarding teat stenotic lesions in “hard milkers”, good outcome of unilateral lesions was referred to the presence of intraluminal small fibrotic septa only, without any damage of the orifice; on the other hand, bilateral lesions might have had a poor outcome due to the damage of their orifices, or due to a genetic cause. This consideration is also supported by Couture and Mulon [21], who observed that injuries to the end of the teat are frustrating to approach, and management of these injuries evolved from being too aggressive when using teat knives, rather than employing a more conservative approach, such as nonreactive teat inserts.

Hyperthelia directly influences mammary health. In does with triplets, the third kid may suckle the non-functional teat with consequent predisposition to mastitis; moreover, the supernumerary teat(s) could hamper the milking [7]. In dairy farms, supernumerary teats are often removed in young animals, and average frequency of infection has been found to be 4.3% in sheep and 29.7% in goats after this surgery [22]. The thelectomies reported in this study were not performed for esthetic reasons, and in all cases have had a good prognosis, as also observed by Edler and Grunert [23], who reported an outcome of 94.6% in 204 teat goat amputations.

Neoplastic lesions on the skin are quite common and are reported both in sheep [9] and goats [24,25]. The majority of cutaneous tumors in sheep are squamous cell carcinoma [26], and the udder is a common site where this neoplasm develops [9]. In the cases here described, removed neoplastic lesions were included in a further study and all were confirmed histologically. The outcome here observed was good, but it is assumed that a good use of a disposable cannula helps to reach good results when the surgery involves the teat. Moreover, simple interrupted sutures are more indicated for surgeries near the teat and its base, due to the excessive traction that continuous sutures may exercise.

Mastectomy is a salvage procedure indicated in many cases, especially in animals with high economic value, at the end of pregnancy, raised as pet or used as embryo donors [14,20,27]. Although El-Maghraby [19] reported that vascular ligation and teat amputation proved to be less traumatic and required less time, effort and expense compared to the classical radical mastectomy, in cases similar to those here reported, where a large area of skin udder is involved, radical mastectomy must be taken into consideration. Even though some authors [14] recommend that creating four semielliptical skin incisions (inverted cloverleaf technique) could better preserve more tissue to reduce excessive skin tension during the closure and consequent dead space formation, in both the cases here described, this was not possible because of the diffuse presence of cutaneous neoplasm, and the chosen alternative was an elliptical incision technique, as also observed in similar cases [20].

The evaluation of intraoperative pain is fundamental and ethically necessary. However, in some field interventions, this practice is difficult to implement and requires the presence of trained professionals [28].

Local anesthetics represent the choice for most operative interventions in ruminants because they do not influence the forestomachal motility [29]. Nevertheless, in some cases, the use of general anesthesia is essential, and the association of an alpha2-adrenergic agonist and dissociative drugs, used here for the sedation of the goats which underwent mastectomy, is also widespread in field anesthesia in small ruminants [6,28]. The tramadol used here represented a good choice as a rescue analgesic drug because it has little influence on both forestomachal motility and the cardiovascular system [29]. Moreover, contrary to the commonly used local anesthetics, tramadol has a quick onset time and a strong efficacy in an acid-inflamed environment, and it is excreted almost entirely with feces and urine [29]. In the present cases, it was selected for intraoperative analgesia due to the presence of inflamed/infected tissues at the surgical site. Under such conditions, the efficacy of local anesthetics may be substantially reduced, as inflammation and infection can alter tissue pH and impair local nerve responsiveness. Systemic administration of tramadol ensured effective analgesia throughout the procedure and complemented local anesthetic techniques, providing reliable pain control even when local anesthetic effectiveness was potentially compromised. This approach allowed safe and adequate intraoperative analgesia while respecting both animal welfare principles and regulatory requirements [29]. Finally, the reason why local anesthesia was performed in the does by mixing physiological solution is because lidocaine 2% can be toxic in this species if used at high doses [30].

Nevertheless, when a surgical procedure is not urgent and a long duration of the surgery is expected, ruminants must be fasted 24 h prior in order to minimize forestomachal distention and the risk of regurgitation during the procedure [19,20].

## 5. Conclusions

To conclude, repair of teat lacerations and teat amputation after traumatisms represent urgent interventions that must be properly carried out, but they are not particularly challenging and do not require deep anatomical and surgical knowledge or a complex surgical set, although basic surgical principles must be applied. Moreover, esthetics or functional thelectomy or skin neoplasm removals are simple and plannable. In the case of bilateral stenotic lesions of the canal, the practitioner should avoid overtreatment and should discourage the farmers from rearing their offspring. Mastectomy requires more technical knowledge but, apart from cases of acute mastitis, it is also often programmable; thus, predisposing the surgery to a good outcome too.

For all these interventions, the owner should be educated and made aware that the animal can often return to production and that these injuries can be treated at a reasonable cost. Indeed, although the interventions here described were performed for free, usually the practitioners perform the majority of these surgeries during their routine flock health plans and the costs applied (mastectomies excluded) are between 10 € and 40 €.

Moreover, here the authors would like to emphasize how the owner compliance, together with timely surgery and appropriate technique, may reduce the need for prolonged post-operative antibiotic treatments in light of the increasing awareness of the antimicrobial stewardship principles.

Finally, the post-operative treatments performed by the owner are the key to a successful intervention regardless of the lesion and the type of surgical technique used.

## Figures and Tables

**Table 1:** Surgical treatment of 135 lesions of teat and udder in 129 small ruminants.

Surgical Condition	Ewes	Does	Animals	Cases
Teat wound reparations	17	2	19	19
Teat fistula reparations	2	-	2	2
Teat cistern curettage	22	-	22	26 *
Thelectomies	2	1	3	5 **
Removals of teat skin neoplasms	14	-	14	14
Removals of udder skin neoplasms	67	-	67	67
Mastectomies	-	2	2	2
Total	124	5	129	135

* Bilaterally in 4 animals; ** bilaterally in 2 animals.

## Data Availability

The original contributions presented in this study are included in the article. Further inquiries can be directed to the corresponding author(s).
